# CNV-PCC: An efficient method for detecting copy number variations from next-generation sequencing data

**DOI:** 10.3389/fbioe.2022.1000638

**Published:** 2022-12-01

**Authors:** Tong Zhang, Jinxin Dong, Hua Jiang, Zuyao Zhao, Mengjiao Zhou, Tianting Yuan

**Affiliations:** ^1^ School of Computer Science and Technology, Liaocheng University, Liaocheng, China; ^2^ College of Clinical Medicine, Shandong First Medical University and Shandong Academy of Medical Sciences, Jinan, China

**Keywords:** copy number variations, next-generation sequencing technology, segmentation, pcc, read depth

## Abstract

Copy number variations (CNVs) significantly influence the diversity of the human genome and the occurrence of many complex diseases. The next-generation sequencing (NGS) technology provides rich data for detecting CNVs, and the read depth (RD)-based approach is widely used. However, low CN (copy number of 3–4) duplication events are challenging to identify with existing methods, especially when the size of CNVs is small. In addition, the RD-based approach can only obtain rough breakpoints. We propose a new method, CNV-PCC (detection of CNVs based on Principal Component Classifier), to identify CNVs in whole genome sequencing data. CNV-PPC first uses the split read signal to search for potential breakpoints. A two-stage segmentation strategy is then implemented to enhance the identification capabilities of low CN duplications and small CNVs. Next, the outlier scores are calculated for each segment by PCC (Principal Component Classifier). Finally, the OTSU algorithm calculates the threshold to determine the CNVs regions. The analysis of simulated data results indicates that CNV-PCC outperforms the other methods for sensitivity and F1-score and improves breakpoint accuracy. Furthermore, CNV-PCC shows high consistency on real sequencing samples with other methods. This study demonstrates that CNV-PCC is an effective method for detecting CNVs, even for low CN duplications and small CNVs.

## Introduction

Genetic variation is prevalent across the human genome and can be classified into various types by different lengths. It should be noted that the heterochromatic parts of the genome are still an issue and are not well covered by next-generation sequencing (NGS). Commonly, small-length variation events fall into two categories: single nucleotide variations (SNVs) and short insertions/deletions (Indels) (Lin et al., 2015). Structure variations (SVs) (Ho et al., 2020) represent large-length variation events. Copy number variations (CNVs), as a type of intermediate-scale SVs (ranges from 1 kb to several Mb), refer to copy number gains or losses over large regions of the genome (Redon et al., 2006). CNVs play a significant role in human genome diversity. Statistically, about 12% of the human genome is impacted by copy number change (Zhao et al., 2013). In terms of the copy number (CN) state of CNVs, low CN (CN of 3–4) duplication events show high numbers in all populations (Veerappa et al., 2015). Meanwhile, multiple studies have shown that CNVs are closely associated with certain diseases (Pös et al., 2021). For instance, CNVs of 1q21.1 is associated with multiple pathologies, including autism, learning disabilities, and schizophrenia (Goh et al., 2018). Symptoms of 3q29 microduplication syndrome are intellectual disability, speech impairment, microcephaly, and dental malformations (Wu et al., 2018). In addition, CNVs have important effects on cancer (Li et al., 2020), Alzheimer’s, and Parkinson’s diseases (Gentile et al., 2021). Therefore, the effective detection of CNVs in the genome holds great importance in both biology and biomedicine.

Traditional detection of CNVs relies mainly on microarray technology. But the microarray technology is limited to the number of probes, and it can only detect the CNVs existed in the reference assembly that designed the probes (Alkan et al., 2011). In recent years, NGS (Goodwin et al., 2016; Zhao et al., 2020) technology has developed rapidly and is widely used for genomic variant detection and clinical genetic diagnosis (Butz et al., 2021). It produces short reads with high resolution and coverage, enabling more accurate identification of breakpoints and discovering new variation events than microarray technology. NGS sequence-based detection methods can be divided into four strategies: pair-end mapping (PEM), split read (SR), read depth (RD), and assembly (AS). The RD-based approach is mainly used to detect CNVs. Its basic idea is that the regions with copy number gains will get a higher read depth compared to the normal regions, while the losses will have a lower read depth. There are lots of methods implemented based on this approach, such as CNVnator (Abyzov et al., 2011), FREEC (Boeva et al., 2011), ReadDepth (Miller et al., 2011), GROM-RD (Smith et al., 2015), and recently released iCopyDAV (Dharanipragada et al., 2018), CNV-LOF (Yuan et al., 2021a), and CNV_IFTV (Yuan et al., 2021b). However, the RD-based approach can only detect rough breakpoints. In contrast, the SR-based approach can reach the resolution of a single base. But the SR-based approach relies heavily on the length of the reads (Zhao et al., 2013). Due to the read length of NGS data being short, split reads may match multiple breakpoints, and it is not suitable for detecting segmental duplication regions (Liu et al., 2020). Combining SR signals with other strategies to accurately identify CNVs is feasible. Delly (Rausch et al., 2012) combines SR and RP strategies, and Lumpy (Layer et al., 2014) uses PEM, SR, and RD signals. All of them achieve more accurate results.

The first step of the RD-based approach is to align the reads in genomic coordinates, then the read depth (RD) signals are obtained by calculating the average read counts in the genomic bin. But the read depth signals have a bias (GC-bias) in regions with higher or lower GC content, so it needs to be normalized according to the GC content in the bin. Segmentation is performed after removing GC-bias. The goal is to cluster adjacent bins with similar RD signals into the same segment. The popular segmentation algorithms include circular binary segmentation (CBS) (Venkatraman and Olshen, 2007), Mean-shift (Comaniciu and Meer, 2002), Hidden Markov model (HMM), and LASSO regression. For example, CNVnator performs segmentation with Mean-shift. It calculates a mean-shift vector for each bin based on the RD signals in the adjacent bins and determines segment breakpoints according to the direction of the vector (Abyzov et al., 2011). This method has high sensitivity and localization accuracy. The segmentation of FREEC is accomplished with LASSO regression. After that, genomic gains and losses are predicted by choosing the allelic content that corresponds to the maximal log-likelihood (Boeva et al., 2011). It can estimate the tumor purity of sequenced samples and can estimate the absolute copy numbers (CN) for the predicted CNVs. iCopyDAV combines CBS and total variation minimization (TVM) algorithms for segmentation, which compensates for the deficiency of CBS in segmenting low-coverage sequences, allowing it to detect a larger range of CNVs with high sensitivity and precision (Dharanipragada et al., 2018).

However, the above segmentation processes are performed on the entire genome (global segmentation) and do not consider local read count variability. The fundamental assumption of the RD-based approach is that the read depth is proportional to the number of copies in the region (Simpson et al., 2010; Yuan et al., 2021b). Restricted by the sequence coverage and interfered from the mapping error, the signal intensities of low CN duplications vary less. In tumor cells, normal tissue contamination further weakens the signal intensity, resulting in low CN duplications being masked by normal regions during segmentation. This scenario is more severe in small CNV events (<10 kb). To avoid this problem, CNV-LOF starts the segmentation from a local perspective. It first divides the target genome into multiple contiguous and non-overlapping regions with the same length, then uses the CBS algorithm to segment each subregion. Finally, each genomic segment is assigned an outlier factor to identify CNV regions (Yuan et al., 2021a). This method shows high sensitivity for low-amplitude CNVs and performs well on low tumor purity data. However, focusing only on localized regions can limit its performance on sequencing data with high tumor purity.

Therefore, it is necessary to develop a new CNVs detection method to address: 1) Affected by limited sequence coverage and mapping errors, the signal intensities of low CN duplications fluctuate less, especially in tumor data. This type of signal is easily smoothed out in global segmentation. Many existing methods cannot detect such CNVs, resulting in lower sensitivity. 2) Compared to larger CNVs, small CNVs (<10 kb) show inconspicuous signals and are more easily to be smoothed by large segments. Although most methods perform well in detecting larger CNVs, identifying small CNVs events is more challenging. 3) The RD-based method has a low breakpoint resolution.

For this, we propose a new CNVs detection method called CNV-PCC (Detection of Copy Number Variations based on Principal Component Classifier). It is a single-sample method, and no control sample is required. Unlike the above segmentation strategies, CNV-PCC operates a two-stage segmentation strategy, that is, a combination of global and local segmentation. First, CNV-PCC uses the CBS algorithm to segment the entire genome. Then, the large-length segments resulting from the CBS algorithm are re-segmented into multiple contiguous subsegments with the specified length. It enhances the ability to identify low CN duplications and small CNVs. Besides the read depth, CNV-PCC introduces SR signals to determine breakpoints, improving the breakpoint resolution. RD signal, GC content, and mapping quality are used as PCC’s inputs to calculate outlier scores. The mapping quality indicates the confidence of a read alignment to this position on the reference sequence, which presents lower levels in the mapping error regions (Li et al., 2008; Dong et al., 2020). In CNV-PCC, data points with a lower mapping quality will receive a high outlier score, which can effectively exclude the interference of mapping errors. We apply CNV-PCC to simulated data and real sequencing samples as well as compare it with several popular methods. The results show that CNV-PCC demonstrates excellent performance in simulated data, and proves its reliability in real samples.

## Methods

### Workflow of CNV-PCC

**FIGURE 1:**
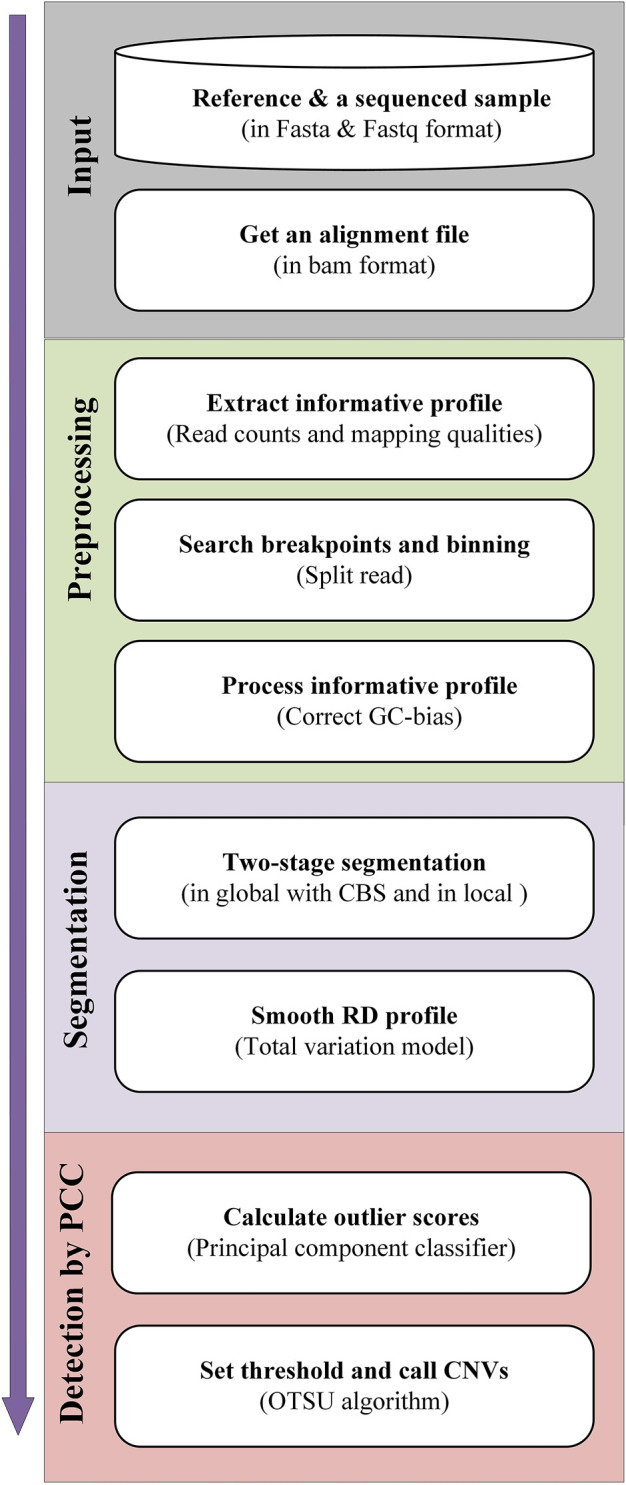
Diagram showing the workflow of the CNV-PCC method. CNV-PCC is composed of four primary parts, including input of BAM files, preprocessing of the informative profile, segmentation, and detection by PCC.

The workflow of the CNN-PCC method is displayed in Figure 1. The alignment file in BAM format is the main input. It is generated with the alignment of sequencing samples (in Fastq format) and reference sequences (e.g., hg38). BWA-MEM approach (Li and Durbin, 2009) completes alignment and is then sorted by SAMTools software (Li et al., 2009). In the preprocessing stage, read counts and mapping qualities are extracted as feature signals. The SR signal is used to locate potential breakpoints. Based on the found breakpoints, the genome is divided into fixed-size bins, and the informative profile is calculated based on the read information in each bin. The RD signals require further normalization to eliminate GC-bias. In the segmentation stage, CNV-PCC implements a two-stage segmentation strategy. It performs segmentation globally and locally, where the global segmentation is achieved using CBS. The local segmentation further subdivides the segments of the CBS division into more subsegments. Meanwhile, the RD signals in the segments are smoothed using the TV algorithm (Condat, 2013) to reduce the noise. In the detection phase, the PCC calculates the outliers for all segments. And the regions of CNVs are identified after the OTSU algorithm determines the threshold. In the following subsections, the principles and implementation of each step are described in detail.

### Preprocessing

The informative profile extracted from the bam file consists of read counts and mapping qualities. The read counts are calculated based on the alignment results, while the mapping qualities can be extracted directly from the bam file. The informative profile requires further preprocessing and includes searching breakpoints, dividing the bins, calculating the feature signals, and correcting the GC-bias.

#### Searching breakpoints and binning

**FIGURE 2:**
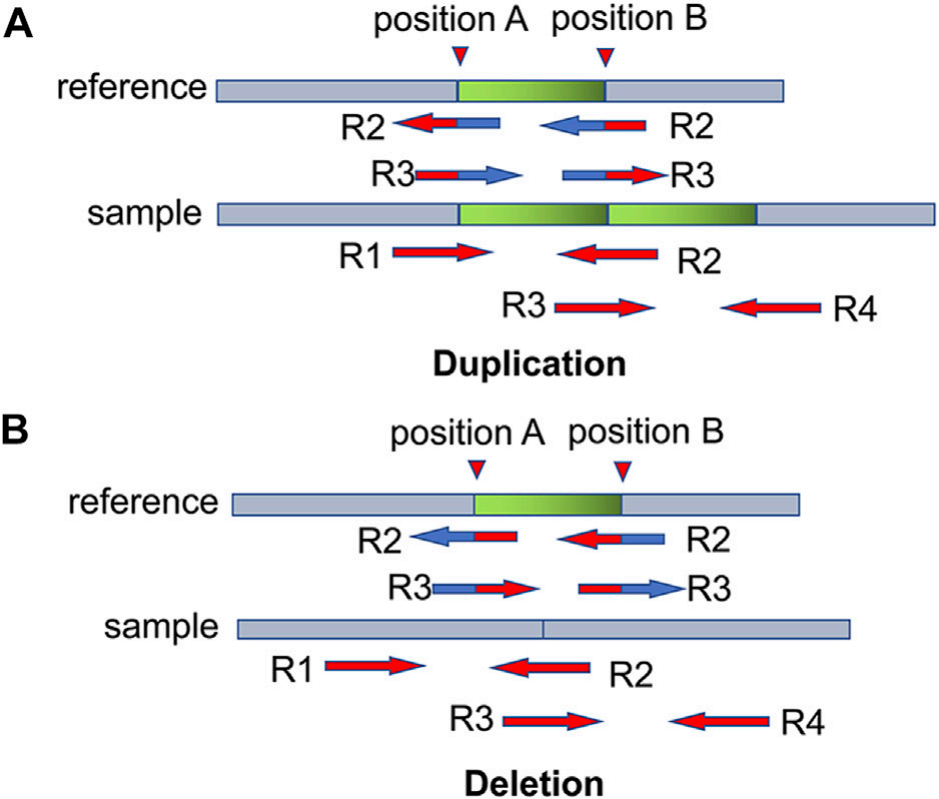
Two examples illustrate the breakpoint information of CNVs contained in split read. **(A,B)** denote duplication and deletion events, respectively. R2 and R3 can be aligned to two positions: position A and position B. The blue part indicates the matched region, and the red part indicates the unmatched region.

Before dividing the bins, CNV-PCC first searches for breakpoints using SR signals. The split reads may contain boundary information of mutation events. For example, in Figure 2A, there are pair-end reads R1 and R2. R1 can match the reference sequence exactly. R2 is located in the boundary region and can only partially match the reference sequence, and this type of read is called split read. The boundary of CNVs can be determined by searching for the breakpoint information in the split read. It can be calculated from the pos field in the BAM file. However, sample sequences usually contain many variation events, and not all split reads are caused by CNVs. Further analysis reveals that reads located at CNVs boundaries can be matched to multiple positions. For example, in the duplication event (Figure 2A), R2 and R3 match both positions A and B. When aligned with the reference sequence, they match two positions: A and B. In the deletion event (Figure 2B), R2 and R3 also match positions A and B. Other mutation events, such as translocation events, may also produce split reads. However, such balance mutation events do not cause fluctuations in the RD signal. Therefore, there is no need to exclude such breakpoints.

Based on the found breakpoints, the entire genome is binned. Bin size (e.g., 1 kb) is first specified. If the distance between two breakpoints is greater than twice the size of the bin, then binning is performed on this region. It is divided into multiple contiguous, non-overlapping bins. After the completion of binning, some smaller bins (<500 bp) will not be favorable for the computation of the RD signal. Therefore, the smaller bins will be merged with adjacent bins (into the previous bin by default) until the size is larger than 500 bp.

#### Calculating the RD and MQ signals of bins

The RD and MQ signals are computed on non-overlapping bins of appropriate size. It can reduce the random fluctuations of read depth caused by noisy signals. For convenience, we use *b*_*i*_ (*i* = 1, 2, 3, ..., *m*) to denote the *i*th bin, and *m* represents the total number of bins. The RD signal for each bin can be calculated *via* Eq. 1.

$$rd_{i}=\frac{\sum_{j=1}^{size\_b_{i}}rc_{j}}{size\_b_{i}}$$ (1)

where $rd_{i}$ denotes the RD value of $b_{i}$ , $rc_{j}$ denotes the read count of the *j*th position in this bin, and $size\_b_{i}$ denotes the size of the $b_{i}$ and is set to 1 kb.

The MQ signals reflect the mean mapping quality level of the reads contained in a bin. The regions with mapping errors present lower values (Lee and Schatz, 2012). In particular, the associated mapping quality is zero when a read is not uniquely mapped to a location (Abyzov et al., 2011). Consequently, a higher MQ value indicates a more confident alignment. The MQ signal of a bin can be calculated by Eq. 2.

$$mq_{i}=\frac{\sum_{j=1}^{size\_b_{i}}mapq_{j}}{size\_b_{i}\cdot r_{i}}$$ (2)

where $mq_{i}$ denotes the MQ value of $b_{i}$ , $mapq_{j}$ denotes the mapping quality of the *j*th position in this bin.

#### Correcting the GC-bias

GC-bias is one of the primary reasons for the inconsistency between the RD signals and sequence coverage (Benjamini and Speed, 2012). The RD values will be biased in regions with low or high GC content. To get representative and accurate RD signals, a common method (Dharanipragada et al., 2018; Liu et al., 2020) is used for correction and the equation below.

$${\tilde{r}}_{i}=\frac{r_{avg}}{r_{gc}}rd_{i}.$$ (3)

Here, $\tilde{r}$ and $rd_{i}$ denote the corrected and original RD value of the *i*th bin $b_{i}$ , separately; $r_{avg}$ denotes the average RD value over all bins, $r_{gc}$ denotes the average RD value for all bins with similar GC content as the $b_{i}$ .

### Segmentation

Segmentation is performed based on the RD profile after GC-bias correction. It consists of two stages: global segmentation, in which the entire genomic region is divided into segments with similar RD values using the CBS, and local segmentation, in which the large segments are re-segmented into contiguous subsegments of the same length. These two phases are briefly analyzed below.

#### Global segmentation with CBS

CBS is a popular segmentation algorithm that is widely used for the detection of CNVs. Its process can be considered as a change point detection problem, whereby finding the location of the bins where the RD value has changed (Venkatraman and Olshen, 2007). CBS performs segmentation on the entire genome, which divides the bins *b*_*1*_, … , *b*_*m*_ into many segments. In each step, it determines a set of consecutive bins *b*_*i*_, *b*_*i+1*_, ..., *b*_*j*_ ( 1≤i < j≤m ). Then utilizing the maximal t-statistic, the mean of the RD values from *b*_*i*_ to *b*_*j*_ is compared with the mean of the remaining bins. If the *p*-value is smaller than the threshold (usually 0.01), it indicates that *b*_*i*_ and *b*_*j*_ (j < *m*) can maximize the test statistic and are viewed as the location of the change point. In other words, the region of *b*_*i*_ to *b*_*j*_ is divided into a segment. The process is applied recursively to the entire genome and divides it into multiple segments.

#### Local segmentation

Once the global segmentation is completed, the local segmentation is further performed on the divided segments. This process can effectively identify CNVs that are smoothed in the large segment, such as low CN duplications and small CNVs. First, the length (Ls) of subsegments is specified. Then the segments with a length greater than Ls are divided into multiple consecutive and non-overlapping subsegments. Each subsegment has the same length Ls, and the last one may be larger than Ls. The size of Ls correlates with the resolution of CNV. Typically, a small Ls will give higher detection resolution and sensitivity but will cause lots of false-positive events. While larger Ls will provide higher precision, the false negatives are hard to control. Users can set the size of Ls according to actual requirements. In our study, the size of Ls is set to 10 kb. After the local segmentation is finished, all segments (both subsegments generated by local segmentation and segments not locally segmented) are arranged sequentially and represented by Eq. 4.

$$RS=\left\{rs_{1},rs_{2},rs_{3},...,rs_{n}\right\},$$ (4)

where $rs_{i}$ denotes the *i*th segment*, and n* denotes the total number of segments.

#### Smoothing the RD profile by total variation

When the segmentation is completed, the RD signals in the segments need to be smoothed and denoised. The noisy data during sorting and segmentation may lead to new errors. The Total Variation (TV) algorithm implements the smoothing process, where the RD signal containing noise shows a high total variance (Condat, 2013). The TV recovers the original signals by reducing the total variance between adjacent segments while preserving the edge information well. The smoothing equation for RD signals is as follows.

$$\min_{\overset{⌢}{r}}\frac{1}{2}\overset{n}{\underset{i=1}{\sum }}\left|{\tilde{rs}}_{i}\right.-{\overset{⌢}{rs}}_{i}{|}^{2}-\lambda \overset{n-1}{\underset{i=1}{\sum }}\left|{\overset{⌢}{rs}}_{i+1}-{\overset{⌢}{rs}}_{i}\right|$$ (5)

where ${\tilde{rs}}_{i}$ and ${\overset{⌢}{rs}}_{i}$ denote the original RD value and the denoised RD value in the $rs_{i}$ , respectively; *n* denotes the number of segments; the former item of the equation represents the fitting error between the original RD value and the denoised RD value, and the latter term is the L1 norm of total variance. λ is the penalty parameter of this term and is used to adjust the constraint size of the total variance. the larger the value of λ, the stronger the penalty. When it tends to infinity, all RD values converge to the same value. When the λ is 0, the original signals are retained. The user can specify the value of λ.

#### Calling CNVs with CNV-PCC

After segmentation, the new signals (RD, GC content, and MQ, in segment units) serve as three features of the PCC for calculating outlier scores. The three features are represented with matrix N, where row vector ***r*** = [*r*_*1*_, *r*_*2*_, ..., *r*_*n*_], ***g*** = [*g*_*1*_, *g*_*2*_, ..., *g*_*n*_], and ***m*** = [*m*_*1*_, *m*_*2*_, ..., *m*_*n*_] denotes the RD, GC content, and MQ signal, respectively; *r*_*i*_ and *m*_*i*_ denote the RD value and MQ value of the *i*th segment, respectively, and they are the mean values of the corresponding signals in the segment. Each column vector (*r*_*i*_, *m*_*i*_, *g*_*i*_)^T^ can be viewed as a sample in PCC.

$$N=\left[\begin{array}{cccc} \begin{array}{c} r_{1}\\ m_{1}\\ g_{1} \end{array} & \begin{array}{c} r_{2}\\ m_{2}\\ g_{2} \end{array} & \begin{array}{c} \ldots \\ \ldots \\ \ldots \end{array} & \begin{array}{c} r_{n}\\ m_{n}\\ g_{n} \end{array} \end{array}\right].$$ (6)

Principal component classifier (PCC) (Shyu et al., 2003) is built on principal component analysis (PCA). PCA is an algorithm commonly used for dimensionality reduction of high-dimensional data. The main principle of PCA is to project the original high-dimensional data onto some low-dimensional space by linear transformation and make its variance as large as possible, so that the valid information of the data can be retained to the maximum. PCA has been applied to the CNV detection problem as a data correction technique (Chen et al., 2011) rather than as the main method for identifying CNVs. Algorithm 1 describes the steps of PCC detection. Its primary objective is to project the three-dimensional matrix *N* onto the one-dimensional vector ***V*** and find the abnormal samples according to the projection distance.

Algorithm 1 Detection of CNVs with PCC.

1: Standardized matrix *N* and denoted by matrix *X*;
2: Calculate the covariance matrix: $=\frac{1}{m-1}XX^{T}$ ;
3: Solve the eigenvalue-eigenvector pairs of C: ( $\lambda_{1}$ , ***e***_1_), ( $\lambda_{2}$ , ***e***_2_) and ( $\lambda_{3}$ , ***e***_3_), $\lambda_{1}\geq \lambda_{2}\geq$ $\lambda_{3}$ ;
4: Calculate the projected distance *d* of each data sample $x_{i}$ on ***e***_*1*_ as an outlier score: $\left(x_{i}\right)=d_{i}=\frac{\sqrt{{\left(x_{i}-e_{1}\right)}^{T}\cdot \left(x_{i}-e_{1}\right)}}{\lambda_{1}}$ , *i* = 1, 2, 3, ..., *n*;
5: Set the threshold *t* with OTSU algorithm; the samples with outlier scores greater than *t* ( $score\left(x_{i}\right)\geq t$ ) are judged as anomalous;
6: Determine the baseline based on the mean RD value and call CNVs.

In step 1, the three features ***r***, ***m***, and ***g*** are normalized to the same scale. This is because the value of MQ is generally larger than the value of RD and GC content, and when projected into the low-dimensional space, the variable MQ will receive a larger weight in the principal component. The two features can be standardized using the following equation:

$$r^{\prime }=\left(r-\bar{r}\right)/r_{sd}$$ (7)

$$m^{\prime }=\left(m-\bar{m}\right)/m_{sd}$$ (8)

$$g^{\prime }=\left(g-\bar{g}\right)/g_{sd}$$ (9)

In Eq. 7, *r′* represents the standardized RD, $\bar{r}$ and $r_{sd}$ represents the mean value and standard deviation of RD, respectively. The normalization process of MQ and GC content is the same as RD and is shown in Eq. 8 and Eq. 9. After standardization, the mean value in each feature turns to 0 and the standard deviation to 1. This ensures that all features have the same influence on the principal component variables. In step 3, the covariance matrix C can be decomposed into orthogonal vectors, called eigenvectors, associated with eigenvalues. The eigenvectors reflect the different directions in which the variance of the sample data changes. The eigenvalues indicate the variance magnitude of the data in the corresponding directions. The eigenvectors ***e***_1_ with high eigenvalues capture most of the data's variance and serve as the principal component vector. In step 4, RD is the main feature to identify CNV. Thus, only the projection distance from the sample to ***e***_1_ needs to be calculated. The outlier score is the weighted Euclidean distance between each sample to the eigenvector ***e***_1_. Samples with larger outlier values indicate potential CNVs or mapping error regions. In step 5, the threshold is set to determine the anomalous samples. The distance projected onto ***e***_1_ varies widely for samples with different sequence coverage. To accommodate data with different sequence coverage, we use the OTSU (Goh et al., 2018) algorithm to calculate the threshold. OTSU is a global binary segmentation algorithm, which is mainly used for the segmentation of grayscale maps. The best threshold obtained maximizes the separability of the resulting gray levels. It dynamically gets a threshold by traversing all the scores in an interval to maximize the variance between the two classes. In this step, we first transform the outlier score into a floating point number with two decimal places. Then, we traverse the outlier scores between the lower 35% quantile and the upper 85% quantile to find the optimal threshold t in increments of 0.01 each time. Samples with scores above t are considered anomalous samples. In step 6, the baseline is defined as the mean RD value of the remaining samples after removing the abnormal samples. Samples with RD values above a quarter of the baseline are considered duplication (gain), and those below a quarter of the baseline are considered deletion (loss) events.

## Results

CNV-PCC software is implemented in python and R languages, and it is freely available at https://github.com/SuphandsomeB/CNV-PCC. For a reasonable performance evaluation of CNV-PCC, we first build a comparison experiment on simulated data. The ground truth possessed by the simulated data guarantees the reliability of the evaluation. We compare the CNV-PCC with five popular methods (CNVnator, FREEC, Delly, CNV-LOF, and CNV_IFTV) concerning the precision, sensitivity, and F1-score. After that, we compare their boundary bias and the size distribution of the identified CNVs. To ensure the fairness of the experiment, we adjust the bin size of certain methods so that they can detect small CNVs. For example, the bin size of the CNVnator is set to the recommended value (90 bp) (Abyzov et al., 2011), and the bin size of FREEC is set to 1 kb. The remaining methods use their default parameters. Subsequently, the real samples are used to verify the validity of CNV-PCC.

### Simulation studies

**FIGURE 3:**
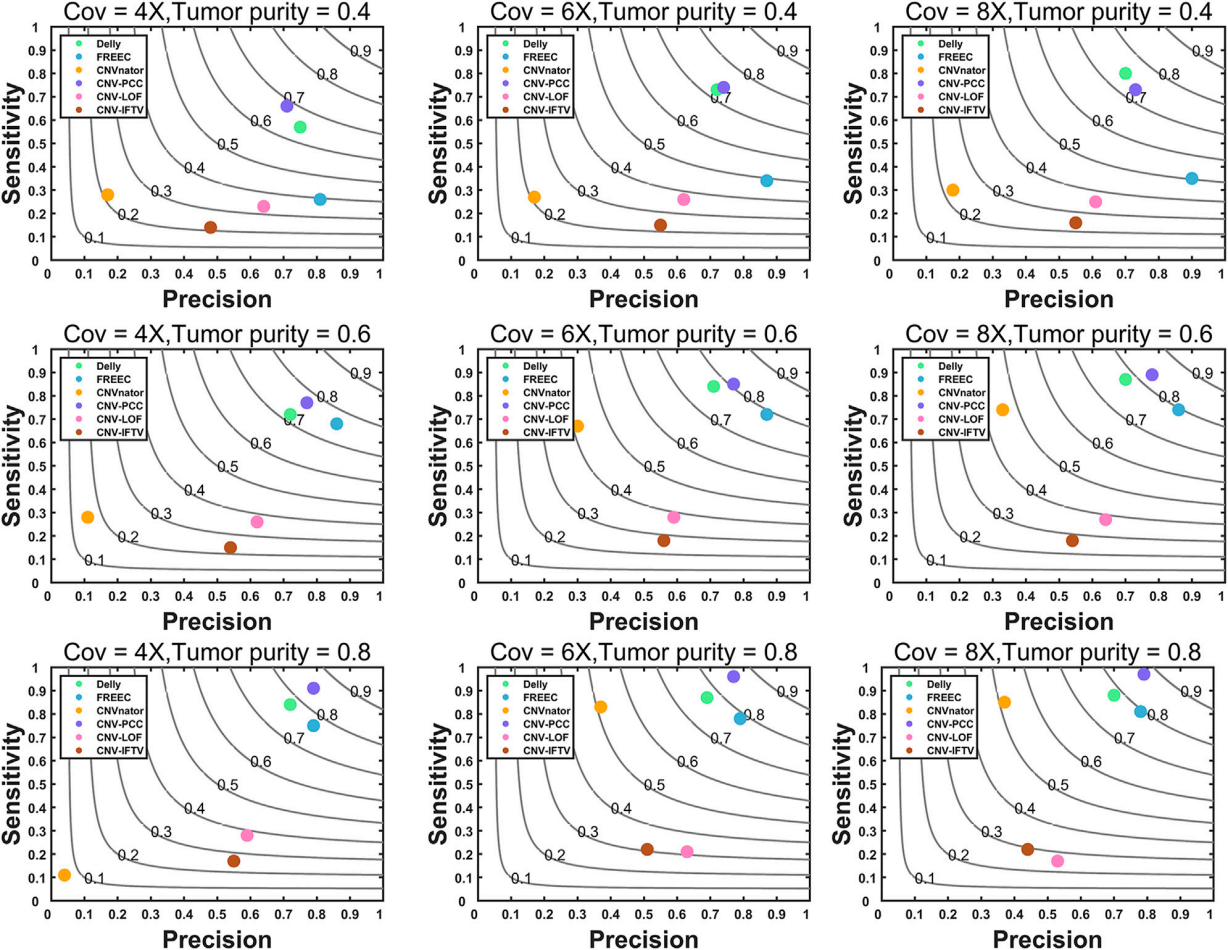
Comparisons of precision, sensitivity, and F1-score between CNV-PCC and five methods (CNVnator, FREEC, Delly, CNV-LOF, and CNV_IFTV). Gray curves indicate F1-score.

The comprehensive simulation software SinC (Pattnaik et al., 2014) and the sequence processing tool seqtk (https://github.com/lh3/seqtk) are used to generate the simulated datasets. All simulated data are generated based on chromosome 21 in the reference genome GRCH38/hg38 (Guo et al., 2017). The coverage is set to 4X, 6X, and 8X. CNV detection is widely used in the field of oncogenetic. To evaluate the performance of each method in this realistic scenario, we simulated tumor purity. The tumor purity is set to 0.4, 0.6, and 0.8, and 30 replicate samples are simulated for each configuration. A total of 26 CNVs are generated in each simulation replication, including 16 duplications and 10 deletions. The variation sizes range from 1kb to 200 kb. To simulate the real situation better, we also generate larger CNVs of size 1–3 Mb. There are 12 CNVs of size 1–10 kb with a frequency of 46.2%, 11 CNVs of size 10–200 kb with a frequency of 42.3%, and three CNVs of size 1–3 Mb with a frequency of 11.5%. The CN of duplications is three and 4. With the generated simulated datasets, CNV-PCC is compared with the five methods. A called CNV is considered a true positive event if there is a 50% reciprocal overlap region between it and the true CNV. The precision, sensitivity, and F1-score are used as metrics in the evaluation, and the results are shown in Figure 3. In the figure, each value of the evaluation metric is the average of 30 simulation replications over each configuration.

CNV-PCC is consistently more sensitive and has a higher F1-score than other methods across almost all coverage levels and tumor purity (Figure 3). In terms of precision, FREEC performs the best among all data, followed by CNV-PCC and Delly. CNV-PCC and Delly are the only methods suitable for detecting all coverages and tumor purity. FREEC and CNVnator are not applicable for detection at low tumor purity. CNV-LOF and CNV_IFTV do not support detecting this type of CNV (They show low metric values on all data). FREEC and CNVnator are more effective in medium and high tumor purity data. CNV-PCC’s superior sensitivity and F1-score are most notable in the high tumor purity data. For example, the sensitivity and F1-score of CNV-PCC at 6X and 8X coverage are 0.96 and 0.86. Compared to 0.88 and 0.78 for Delly, 0.81 and 0.79 for FREEC, and 0.85 and 0.72 for CNVnator.

**FIGURE 4:**
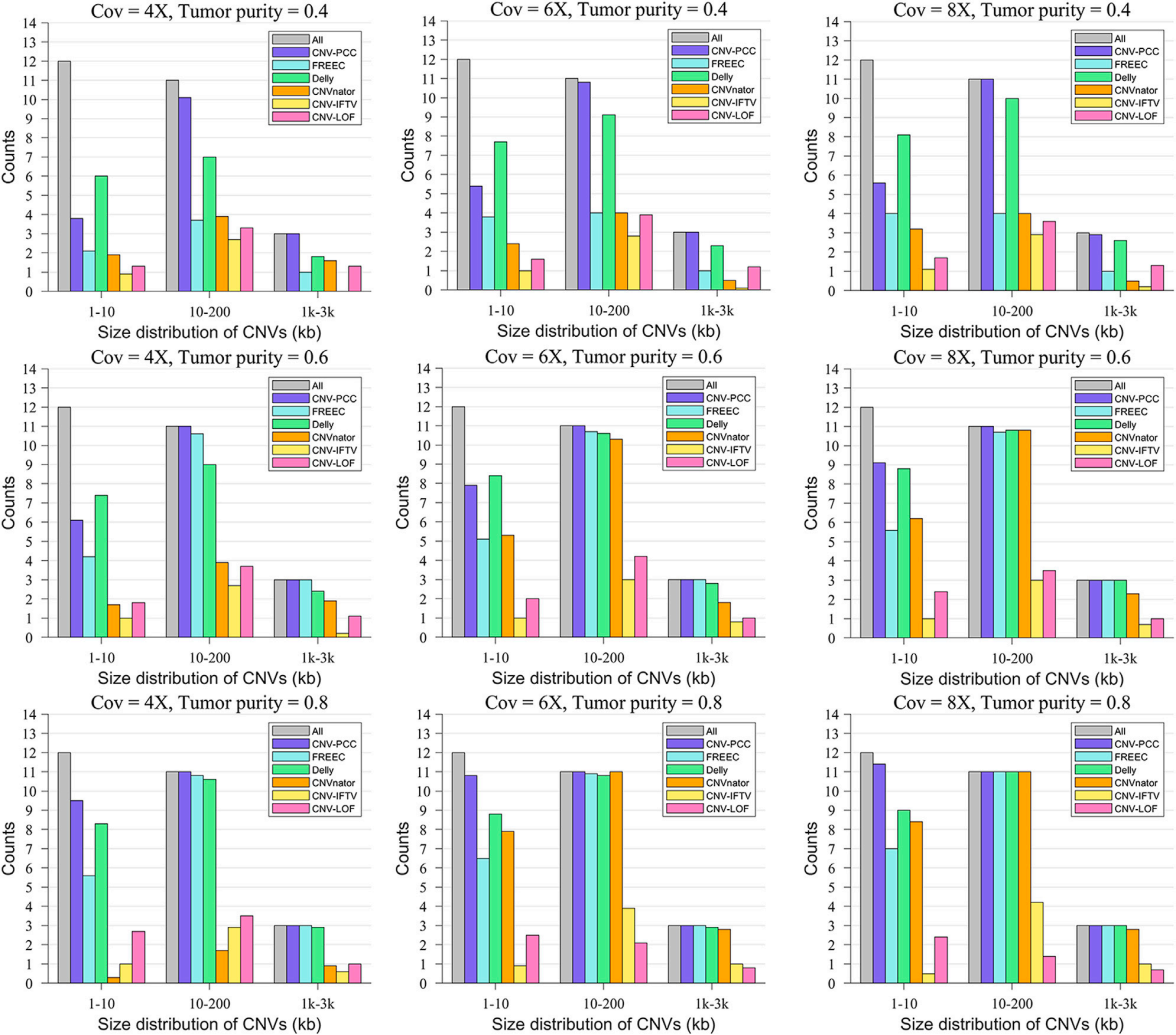
Comparisons of the size distribution of CNVs between CNV-PCC and five methods (CNVnator, FREEC, Delly, CNV-LOF, and CNV_IFTV). The gray bars indicate the number of all CNVs in this range.

Figure 4 shows the size distribution of CNVs detected by the six methods. The gray bars indicate the number of all CNVs in this range. In terms of the number of small CNVs (1–10 kb) detected, Delly detects the most on the low tumor purity data, followed by CNV-PCC. All RD-based methods (all methods except Delly) exhibited low performance. It is because small CNVs have insignificant RD signal changes at low tumor purity and are easily smoothed by adjacent segments. The performance of all methods (except CNV-LOF and CNV_IFTV) improved as the tumor purity increased. On the high tumor purity data, CNV-PCC always identifies the highest number of small CNVs. It is around 2 higher than the second-ranked Delly.

**FIGURE 5:**
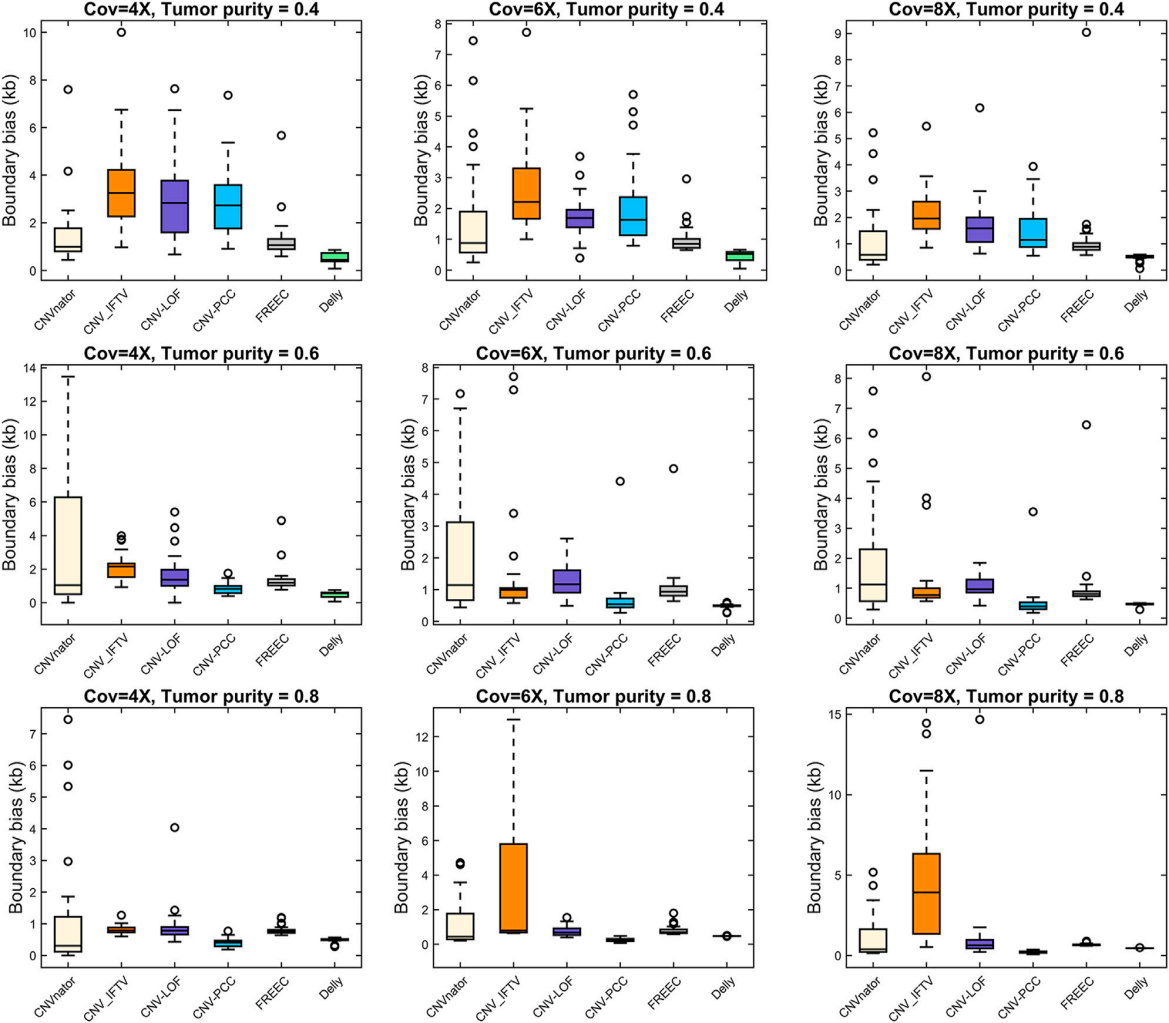
Comparisons of boundary bias between CNV-PCC and five methods (CNVnator, FREEC, Delly, CNV-LOF, and CNV_IFTV).

The boundary bias of each method is shown in Figure 5. Here, we are not counting the boundary bias at larger CNVs (1–3 Mb) because it may yield bigger values that are not favorable for comparison. Delly performs best in the low tumor purity data. Except for Delly, all methods exhibit large boundary bias in the low tumor purity data. With increasing tumor purity and coverage, the boundary bias gradually decreased. In high tumor purity, CNV-PCC reaches the optimal boundary bias, lower than other RD-based methods, and lower than Delly in the 6X and 8X coverage.

Collectively, CNV-PCC shows the highest sensitivity and F1-score on almost all data. Regarding the size of CNVs detected, Delly detects more at low tumor purity for small CNVs, while CNV-PCC works well at high tumor purity. A similar situation is observed for boundary bias. It is because the RD-based methods mainly rely on the RD signal to identify CNVs. Small CNVs (especially low CN duplications) have less RD signal change at low tumor purity and are easily smoothed by adjacent segments. Meanwhile, the SR signal is relatively less at low tumor purity (especially at low coverage), which leads to high boundary bias and imprecise breakpoint identification in CNV-PCC. For large CNVs, CNV-PCC is consistently more sensitive than other methods. Taken together, CNV-PCC is an effective method for detecting CNVs.

### Real data studies

**FIGURE 6:**
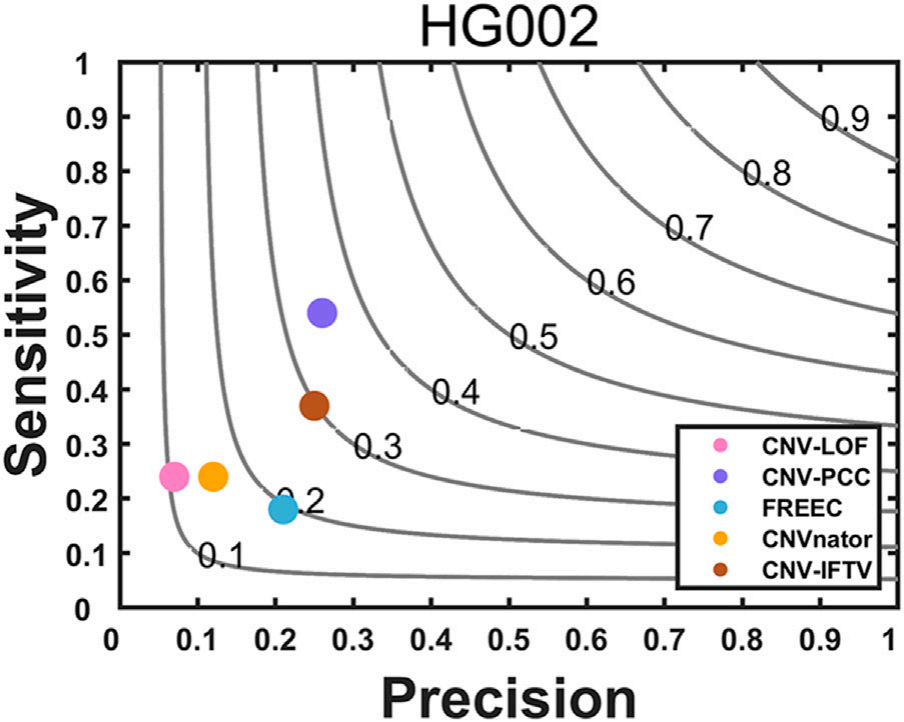
Comparisons of precision, sensitivity, and F1-score between CNV-PCC and four methods (CNVnator, FREEC, CNV-LOF, and CNV_IFTV) on HG002.

In terms of real data, we chose sequencing samples (HG002) from the son of the Ashkenazim Jewish (AJ) trio. There is an amount of publicly available data published by the Genome in a Bottle (GIAB) Consortium (Zook et al., 2016). We select a short read-based dataset to evaluate the performance of CNV-PCC and compare it with four existing methods (CNVnator, FREEC, CNV-LOF, CNV_IFTV). The benchmark generated by the Genome in a Bottle (GIAB) consortium can be used to evaluate the performance of each method. The results of the comparison of sensitivity, precision, and F1-score for the five methods are shown in Figure 6.

From Figure 6, it can be seen that CNV-PCC has the highest F1-score, which is 5% higher than the next-best method CNV_IFTV (35% *versus* 30%), with FREEC at 19%, CNVnatorat at 16%, and CNV-LOF at 11%. In addition, CNV-PCC outperforms the other methods in terms of precision and sensitivity. However, the metric values of each method are relatively low compared to the simulated data. This is due to the variant distribution being more complex in the real genome (Hyman, 2021). The read depth signal in some regions is affected and deviates from the true value, resulting in false positive or false negative events.

**FIGURE 7:**
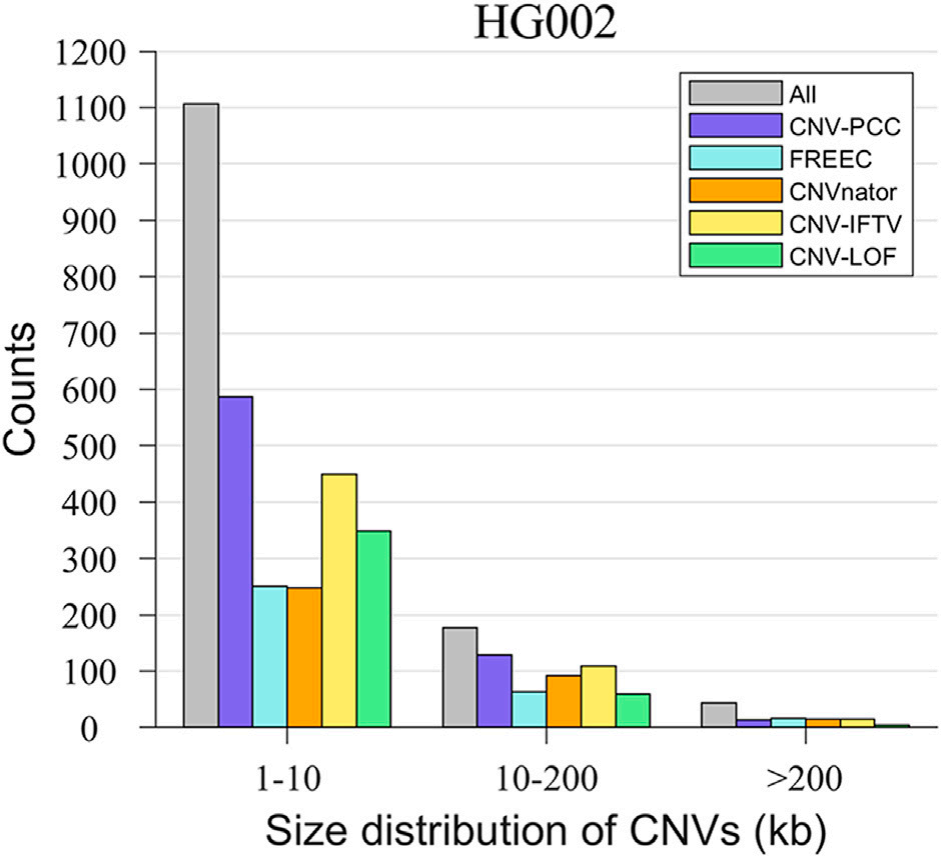
Comparisons of the size distribution of CNVs between CNV-PCC and four methods (CNVnator, FREEC, CNV-LOF, and CNV_IFTV) on HG002. The gray bars indicate the number of all CNVs in this size.

A unique advantage of CNV-PCC over other methods is its greater sensitivity to small CNVs. To demonstrate this capability, we show the size distribution of CNVs detected by the five methods in Figure 7. The delineation of the size interval of CNVs is roughly consistent with that in the simulated data, and the gray bars indicate the number of all CNVs within this range. The number of small CNVs (1–10 kb) is the largest, accounting for 83.3%. Medium CNVs (10–200 kb) and large CNVs (>200 kb) are 13.3% and 3.4%, respectively. As expected, CNV-PCC detects the highest number of small CNVs (587), followed by CNV_IFTV (450) and CNV-LOF (349). The number of small CNVs detected by FREEC and CNVnator is essentially the same (251 and 248). Concerning medium CNVs, CNV-PCC also has the highest number of identifications (129). The rest are, in order, CNV_IFTV (109), CNVnator (92), FREEC (63), and CNV-LOF (60). In terms of the recognition ability of large CNVs, except for the poor performance of CNV-LOF, the remaining four methods recognized essentially the same number (∼15).

## Conclusion

In this paper, we propose CNV-PCC, a CNVs detection method applied to whole genome sequencing data from short read sequencers. CNV-PCC takes bam files as input and extracts RD, GC content, and MQ signals to identify the regions of CNVs. Compared with existing methods, it has three new features as follows: 1) CNV-PCC uses the PCC model to detect CNVs, and the sensibility of PCC to feature signals change makes it effective in detecting low CN duplications. 2) CNV-PCC performs segmentation globally and locally. Compared with the strategy of single segmentation, it effectively avoids the problem of low CN duplications and small CNVs being smoothed. 3) CNV-PCC uses SR signals to find breakpoints, significantly reducing boundary bias.

We first test the performance of CNV-PCC in simulation experiments with different configurations, and compare it with five popular methods in terms of precision, sensitivity, and F1-score. The results show that CNV-PCC gets the highest sensitivity and F1-score in almost every configuration, demonstrating its excellent performance. From the size distribution and boundary deviation of detected CNVs, CNV-PCC is slightly inferior to Delly for low tumor purity data. With increasing tumor purity, the performance of CNV-PCC improved significantly. It surpassed Delly in the high tumor purity data, both in terms of the number of small CNVs detected and the breakpoint accuracy. Next, the performance of CNV-PCC is compared with the four methods on a real sample (HG002). The result shows that CNV-PCC has the highest F1-score, which is better than the other four methods. Moreover, CNV-PCC identifies the greatest number of CNVs (including small CNVs), proving that our method is reliable.

Currently, the shortcomings of the CNV-PCC are mainly reflected in two aspects. First, the bin size is fixed at 1 kb, which is the maximum resolution of CNVs detected by CNV-PCC. It is difficult to detect some smaller CNVs (<1 kb) even if the SR signal can identify the breakpoints. Meanwhile, CNV-PCC performs poorly in identifying small CNVs on low tumor purity data. Second, CNV-PCC applies to WGS data and has not been developed for identifying CNVs in WES. In future work, we plan to enhance the role of SR signal and reformulate the binning strategy for solving the identification problem of small CNVs. It can reduce the effect of tumor purity. Secondly, extending the CNV-PCC to enable its use for identifying WES-based CNVs.

## Data Availability

The original contributions presented in the study are included in the article, further inquiries can be directed to the corresponding author.
